# Hubert Liston Willcox

**Published:** 1913-12

**Authors:** 


### Obituary.

### HUBERT LISTON WILLCOX.

The death of Mr. Hubert Liston Willcox, of Warminster, at the early age of 36, occurred on Dec. 3rd, and was due to blood poisoning, resulting from a finger being scratched while assisting at an operation for acute peritonitis. He was educated at King's College Hospital, London, and took the qualifications of L.R.C.P. and M.R.C.S. in 1901.

For the untimely loss of his son under such singularly sad circumstances, we extend our heartfelt sympathy to his father, Dr. R. Lewis Willcox, with whom he was in practice at Warminster, to his widow, mother, and other members of the family. Mr. Willcox had a peculiarly attractive personality, and while commanding the confidence of many of the best families in the county, he was equally popular with his poorer patients, who always felt that he was a friend who would never spare any effort on their behalf, and practically the whole town of Warminster and the surrounding district mourned his death.

